# Engineered Dutasteride-Lipid Based Nanoparticle (DST-LNP) System Using Oleic and Stearic Acid for Topical Delivery

**DOI:** 10.3390/bioengineering9010011

**Published:** 2022-01-01

**Authors:** Norhayati Mohamed Noor, Sana Umar, Azila Abdul-Aziz, Khalid Sheikh, Satyanarayana Somavarapu

**Affiliations:** 1 Department of Pharmaceutics, UCL School of Pharmacy, University College London, London WC1N 1AX, UK; sana.umar.13@alumni.ucl.ac.uk (S.U.); k.sheikh@ucl.ac.uk (K.S.); 2 Cosmeceutical & Fragrance Laboratory, Institute of Bioproduct Development (N22), Universiti Teknologi Malaysia, Johor Bahru 81310, Malaysia; azila@ibd.utm.my; 3 Department of Chemical and Environmental Engineering, Malaysia-Japan International Institute of Technology, Universiti Teknologi Malaysia, Kuala Lumpur 54100, Malaysia

**Keywords:** alopecia, dutasteride (DST), lipid nanoparticle (LNP), topical delivery, oleic acid, stearic acid

## Abstract

Male pattern baldness (MPB) is a common condition that has a negative impact on the psycho-social health of many men. This study aims to engineer an alcohol-free formulation to cater for individuals who may have had allergic reactions to alcohol-based preparations. A lipid-based nanoparticle system composed of stearic and oleic acid (solid and liquid lipid) was used to deliver dutasteride (DST) for topical application. Two compositions, with oleic acid (Formulation A) and without (Formulation B), were compared to analyse the role of oleic acid as a potential active ingredient in addition to DST. DST-loaded LNP were prepared using the emulsification–ultrasonication method. All of the prepared formulations were spherical in shape in the nanometric size range (150–300 nm), with entrapment efficiencies of >75%. X-ray diffractograms revealed that DST exists in an amorphous form within the NLP matrices. The drug release behaviour from both LNP preparations displayed slow release of DST. Permeation studies through pig ear skin demonstrated that DST-LNP with oleic acid produced significantly lower permeation into the dermis compared to the formulation without oleic acid. These results suggest that the proposed formulation presents several characteristics which are novel, indicating its suitability for the dermal delivery of anti-androgenic molecules.

## 1. Introduction

Male pattern baldness (MPB), also known as androgenic alopecia (AGA), is a chronic disorder and the most frequent kind of hair loss in males, with a prevalence of 20% at the age of 20–30 and a 10% annual increase in incidence [1,2,3]. The pathophysiology has a close genetic association, whereby affected individuals have increased sensitivity to androgens. Though androgens are capable of regulating and stimulating hair growth, in affected individuals, they reduce the growth (anagen) phase of the hair follicle, leading to miniaturisation and eventually transforming them to tiny vellus hair follicles. Testosterone is one of the key androgens involved; it is converted to its potent form, dihydrotestosterone (DHT), by the 5-α reductase enzyme, which appears to have a higher activity on balding scalp compared to nonbalding scalp [4]. The psychological and social effects of baldness can be considerable, particularly in younger men, who are more likely to feel anxious about appearing older due to their hair loss. [5,6]. Currently, considerable research on hair growth molecules, either from plant derivatives or synthetic chemicals, is being conducted [7,8,9]. However, there are only two approved treatments so far: Minoxidil, which is the only topical application available, and finasteride (type II 5α-reductase inhibitor—taken orally), which is available in the form of tablets [10]. 

Dutasteride is a type I and type II 5-reductase inhibitor that is three times more effective than finasteride at inhibiting type II enzyme action, and 100 times more potent at inhibiting type I enzyme action than finasteride [11]. Another study found that, dutasteride produced approximately 20% more effective at decreasing serum DHT level than finasteride [10]. Dutasteride is approved at a dose of 0.5 mg level as a treatment for benign prostatic hyperplasia (BPH). A randomised placebo-controlled study also demonstrated that dutasteride at the same dose was more rapid at promoting hair growth than finasteride [10]. 

Topical administration of dutasteride might be a more appropriate drug-delivery approach to lessen the systemic consequences of decreasing DHT levels, such as impaired sexual desire, increased depression, and ejaculation difficulties [12,13]. There is also the need for a nonethanol based medium of delivery, as cases of contact dermatitis and irritation can be associated with this, as observed with minoxidil. Thus, a formulation with dutasteride in a lipid-based nanoparticle system such as a nanostructured lipid carrier (NLC) would be advantageous for dermal application. The solid lipid matrix of NLC allows a controlled release profile for many substances. Their film forming property also allows for drug occlusion, in some cases enabling it to be supplied over a prolonged period, thus reducing systemic absorption [14]. The less ordered liquid lipid present in the NLC system matrix allows for greater therapeutic drug loading. Their small size also ensures close contact to the stratum corneum, increasing drug penetration into the skin. In addition to this, the physiological and biodegradable composition of lipids exhibits low toxicity, which enhances tolerability. A previous study found that a dutasteride loaded NLC system reduced cytotoxicity on the hair follicle dermal papilla cells compared to dutasteride alone [15]. Furthermore, the poorly water-soluble property of dutasteride (0.038 ng/mL; Log P = 5.09) makes lipid-based nanoparticle systems useful as potential carriers. 

The aim of this study was to formulate dutasteride loaded into a lipid-based nanoparticle system, using solid and liquid lipids for topical delivery. Stearic acid was chosen as the solid lipid and oleic acid as the liquid lipid, as oleic acid showed potent anti-androgenic activity in a previous study [16]. Liu et al. [16] found that unsaturated fatty acid (oleic acid and α-linolenic acid) showed a proliferation inhibitory effect on lymph-node carcinoma of the prostate (LNCaP) cells, suggesting that fatty acids with 5α-reductase inhibitory activity block the conversion of testosterone to 5α-dihydrotestosterone (DHT). Rashed et al. [17] found that sesame oil (containing 66.7% of oleic acid) produced a reduction in prostate weight and a significant reduction in the testosterone levels of castrated rats, suggesting that the plant could be used as new potential therapeutic candidate for the treatment of androgen-related diseases. This combination of components could produce a formulation in which dutasteride, stearic and oleic acid act together to have a synergistic effect in preventing hair-loss and promoting hair growth activity. 

## 2. Materials and Methods

### 2.1. Materials

Stearic and oleic acid were purchased from Tokyo Chemical Industry (Oxford, UK). Dutasteride (purity > 98.0%) was obtained from Carbosynth (Compton, UK). Ethanol (96% *v*/*v* analytical grade) and Sephadex G-50 was obtained from Sigma-Aldrich (Dorset, UK). Phosal^®^ 53 MCT and Lutrol^®^ micro 68 were supplied by Lipoid GmbH (Ludwigshafen, Germany) and BASF Group (Ludwigshafen, Germany), respectively. Water (HPLC grade) was purchased from Fisher Scientific (Loughborough, UK). Deionised water was prepared in-house (PURELAB, ELGA, London, UK). 

### 2.2. Preparation of Dutasteride-Loaded Nanostructured Lipid Carriers (DST-NLP)

DST-LNP were prepared by the emulsification–ultrasonication method as described by a previous study [15]. Dutasteride, stearic acid, oleic acid and Phosal^®^ 53 MCT were weighed in a glass vial. Lutrol^®^ micro 68 with water (10 mL) was added into another glass vial. Both vials were heated in a water bath at 80–90 °C, independently. The drug was dissolved in the liquid lipid (with continuous magnetic stirring), and then the aqueous solution was added into the oil-phase solution. The mixture was homogenised using an IKA Ultra Turrax T25 (IKA Werke, Staufen, Germany) at 19,000 rpm for 10 min. The hot solution was further processed using a probe-type sonicator (MSE Soniprep 150, MSE, Loughborough, UK) at 18 W, for 5 min. Then, 2 mL of the hot dispersion was syringed (needle: 25 gauge, 5/8th inch) into 10 mL of cold water (4–8 °C) and stirred for 10 min to obtain nanoparticles. The formulation was stored in a refrigerator (4–8 °C) before being characterised. 

### 2.3. Experimental Design of Preparation of DST-LNP 

The amount of solid lipid, surfactant and liquid lipid used to prepare DST-LNP was varied to obtain particles with a suitable mean particle size and narrow size distribution, as well as high entrapment efficiency. This was achieved by altering the ratios of all the components. Nineteen different preparations, each produced in triplicate, were prepared. 

### 2.4. Characterisation of DST-LNP

#### 2.4.1. Measurement of Particle Size Distribution and Zeta Potential 

The size distribution and zeta potential of nanoparticles without dilution were obtained as Z_Ave_ hydrodynamic diameter, polydispersity index (PDI) and zeta potential (ξ) using a Zetasizer Nano ZS (Malvern Instruments, Malvern, UK) after 1 day, at 25 °C. To this end, 1 mL of the sample was pipetted directly into the zeta potential DTS1070 folded capillary cell (Malvern, UK) without dilution. Measurements were performed three times, and mean values were taken. Zeta potential was calculated from electrophoretic mobility using the Helmholtz-Smoluchowski equation in the Malvern data analysis software.

#### 2.4.2. Determination of Nanoparticle Morphology and Crystallinity 

The morphology of the nanoparticles was determined using transmission electron microscopy (TEM; Philips/FEI CM120 Bio Twin, FEI, Eindhoven, The Netherlands). Samples were placed on copper grids for viewing and excess droplets was removed with filter paper. After 2 min, a drop of 2% uranyl acetate acid was placed onto the copper grid for negative staining and dried at room temperature. 

The crystallisation behaviour between dutasteride and other materials used in the formulation was determined using an XRD. Freshly prepared nanoparticles, with and without dutasteride, were freeze dried. An X-ray diffractometer (Rigaku MiniFlex 600, Rigaku, Tokyo, Japan) equipped with a 600 W X-ray tube, a copper anode operating in reflectance mode at wavelength kα λ 1.5418 Å, voltage of 40 kV and current mA was used for diffraction studies. Samples of dutasteride alone, physical mixture and DST-LNP were mounted to the horizontal axis to obtain a scanning range of 5–40° 2θ at a step size of 0.05°/min and step rate of 5 step/min at 25 °C

#### 2.4.3. Entrapment Efficiency 

Entrapment efficiency was calculated as described previously [15]. The amount of drug entrapped was determined using Sephadex^®^ gel G-50 as a minicolumn. The presoaked gel was packed in a 5 mL syringe, placed in a tube (sized 50 mL) and centrifuged at 1000 rpm (angle rotor 19776-H, at 104× *g*) for 10 s in a refrigerated centrifuge (Sigma Laborzentrifugen, Osterode, Germany) at 20 °C. Then, 0.5 mL DST-NLP preparation was pipetted in the column and separated at 1000 rpm for 5 min. The column was washed with 0.5 mL deionised water for another 5 min and the product (dutasteride entrapped in LNP) was dissolved using bath sonication in 10 mL of ethanol before being filtered using a 0.22 μm pore size syringe filter (Merck Millipore, Dublin, Ireland). The ethanolic solution was assayed using a validated method of HPLC with a UV/Vis detector (Agilent 1100 Series, Agilent Technologies, Santa Clara, CA, USA) at a wavelength of 241 nm to determine the total and entrapped dutasteride in the DST-LNP [18]. A Synergi™ 4 μm Polar-RP 80 Å, 250 × 4.6 mm column was used as the stationary phase. A ratio of 70:30 (by volume) acetonitrile and 0.1% trifluroacetic acid (TFA) in HPLC grade water was used for the mobile phase. A 1 mL/min flowrate and 30 μL injection volume was applied. 

Encapsulation efficiency was calculated from the amount of entrapped (n_1_) and total dutasteride in the preparation (n_2_) (Equation (1)).

Entrapment efficiency (%) = (n_1_/n_2_) × 100 (1)

where

n_1_ = concentration of entrapped dutasteride in DST-LNP
n_2_ = total concentration of dutasteride in DST-LNP

### 2.5. Physical Stability Study 

The physical stability of the lipid nanoparticles was assessed to explore potential changes in the size distribution, surface charge and entrapment efficiency with time. The formulation was kept in a refrigerator (4–8 °C). Particle size distribution, zeta potential and entrapment efficiency were compared at day 1, 8, 43 and 50. 

### 2.6. In Vitro Drug Release 

In vitro release was evaluated using a Franz diffusion cell (PermeGear, Hellertown, PA, USA) with 0.45 μm cellulose membrane (Merck Millipore, Dublin, Ireland) mounted in the Franz diffusion cell and maintained at 37.0 ± 0.5 °C and stirred using magnetic stirrer (600 rpm) [18]. To this end, 2% sodium dodecyl sulphate (SDS) was added, due to the limited solubility of dutasteride in phosphate-buffered saline (PBS). Formulations A and B, were pipetted into the donor chamber separately, and 200 μL was taken at 0, 2, 4, 6, 8, 24, 28, 32, 36 and 48 h from the receptor chamber and replaced with 200 μL fresh buffer. Samples were injected into the HPLC to determine dutasteride released.

### 2.7. In Vitro Permeation Study 

In vitro permeation was performed using a vertical Franz diffusion cell (PermeGear, Hellertown, PA, USA) in order to mimic topical application of the formulation as described in previous study [15]. Pig ears were obtained from a local slaughterhouse (Farnborough, UK) from freshly slaughtered pig (used for food consumption). The pig ear skin was washed and excised using a scalpel and forceps, and the subcutaneous tissue was removed. The average thickness of the skin was ~0.5 mm. The skin was cut and frozen (−20 °C) for future use. (The diffusion surface area = 0.64 cm^2^). The skin was placed between the donor and receptor chambers and left for equilibrium for 1 h. Then, 5 mL PBS (pH 7.4) with 2% SDS and 0.02% sodium azide (preservative) was filled in the receptor chamber and maintained at 37 °C. Next, 250 μL of Formulations 17 and 19 were pipetted separately into the donor chambers; 200 μL samples were taken at 0, 2, 4, 6, 8, 24, 28, 32, 36 and 48 h, and 200 μL fresh buffer was added to the receptor chamber. The stratum corneum was removed using a tape stripping method after 48 h. The epidermis/dermis was cut into pieces, stirred for 24 h with ethanol, and sonicated for 1 h. The sample was filtered and injected into the HPLC to quantify the drug permeation of the skin. 

### 2.8. Statistical Analysis 

All data were analysed either using a *t*-test or one-way ANOVA and Tukey’s post-hoc test, using IBM SPSS Statistic 22, A *p*-value of less than 0.05 was considered significant.

## 3. Results

### 3.1. Preparation and Characterisation of DST-LNP

**Figure 1:**
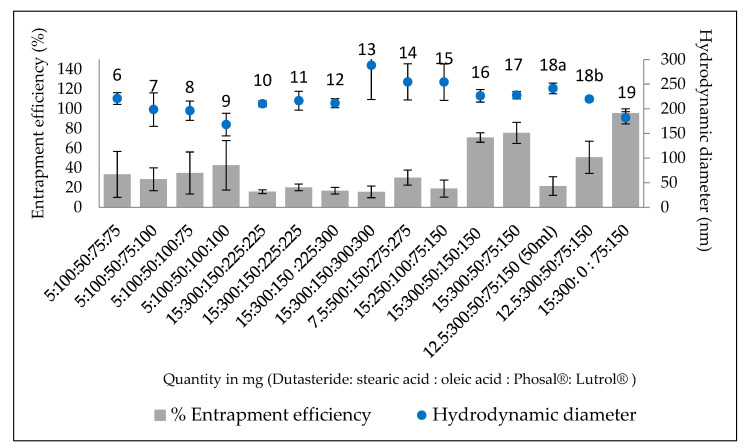
Entrapment efficiency (%EE) and hydrodynamic diameter size of LNP with altered ratios of components for Formulations 6–19.

Formulations 1–19 were produced in ascending numerical order in order to determine which formulation had the optimal characteristics, in particular the highest associated entrapment (Figure 1). The first five formulations showed visible aggregation (data not shown), and were not considered for further analysis. Formulations 10–13 were an upscale of formulations 6–9 by a factor of three, in order to improve the precision of the results (lower than LOQ using HPLC). A clear reduction in the entrapment efficiency was observed due to this upscale. No significant differences (*p* > 0.05) were observed in the entrapment efficiencies of Formulations 10–13. 

All formulations produced particles sized in the range 150–300 nm, with varying levels of entrapment. Formulation 17 was found to have the highest entrapment efficiency (75.5% ± 10.7) among the formulations containing oleic acid. LNP in formulation 17 had an average size of 227.6 ± 7.4 nm. Its composition had the smallest amount of liquid lipid (Phosal^®^ and oleic acid) and the greatest amount of surfactant (Lutrol^®^ micro 68), which may have been the reason for its high entrapment. This demonstrates that a high level of liquid lipid could possibly be associated with a reduction in stability of the particles. In order to optimise this further, a reduction in drug quantity was applied, with the reasoning that by reducing the drug content in the LNP, the %EE would increase. The formulation was carried out in 50 ml and 60 ml (as for the rest of the formulations) quantities with Formulations 18a and 18b, respectively. However, a reduction in %EE was observed instead of an increase.

In order to test the effect of the liquid lipid, in particular, oleic acid, we eliminated oleic acid in Formulation 19. An interesting observation was made with this formulation, namely, the %EE obtained was 96.7% ± 4.4, and the size was 181.8 ± 12.4 nm. Thus, there was a significant increase and decrease in entrapment and size, respectively. This was because oleic acid was added without changing the quantity of the surfactant which controls the particle size and the stability of the dispersion. This is also in line with a previous study, which found that an increase in oleic acid concentration led to a decrease in entrapment efficiency and an increase in size [19]. A possible reason for this is that the increased liquid lipid was not accommodated by the solid lipid during solidification, and was dispersed in the external aqueous phase. The drug may have been partitioned into this liquid lipid, due to its solubility therein and lack of solubility in water. Thus, the formulation with 15 mg of dutasteride, 300 mg of stearic, 75 mg of Phosal^®^ and 150 mg of Phosal^®^ was used in further experiments, with Formulation 17, as well as without oleic acid (Formulation 19). (From this point, Formulation 17 will be called “Formulation A” (with oleic acid) and Formulation 19 “Formulation B” (without oleic acid)).

#### 3.1.1. Determination of Nanoparticle Morphology and Crystallinity

**Figure 2:**
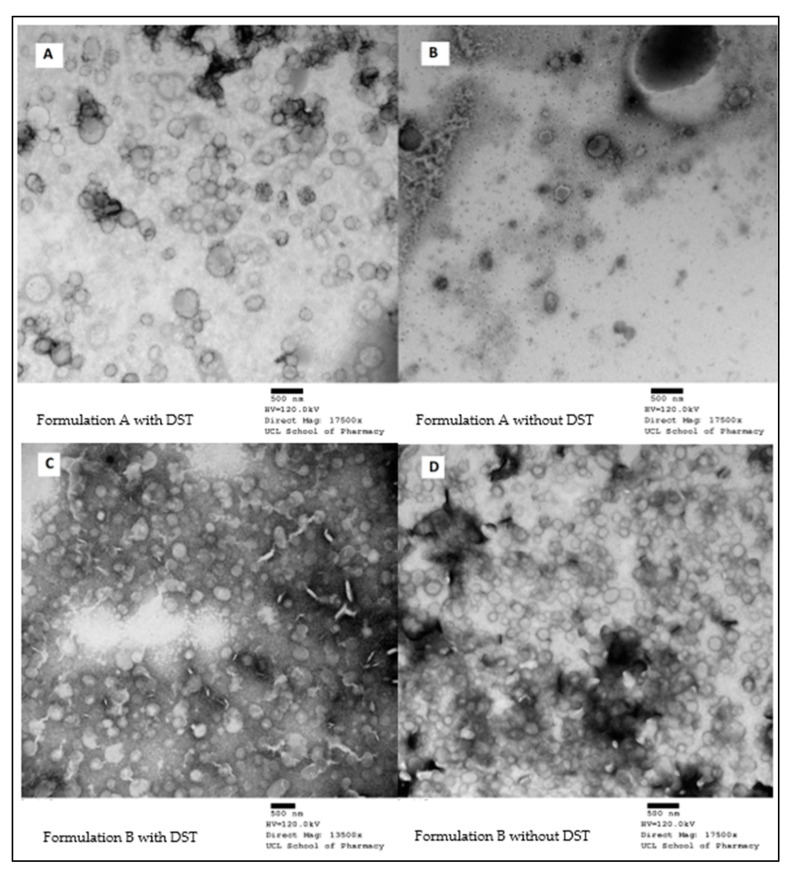
TEM micrographs of Formulation A with dutasteride (**A**), and without dutasteride (**B**) and Formulation B with dutasteride (**C**), and without dutasteride (**D**).

Based on the TEM images (Figure 2), both formulations that consisted of DST showed nonaggregated LNP which were spherical in shape. The size range observed was in agreement with the sizes obtained from the Zetasizer result. 

Formulation A (Figure 2A) showed a greater diversity in size compared to Formulation B (Figure 2B). This is reflected by the PDI of 0.17 ± 0.02 and 0.13 ± 0.01, respectively. The addition of dutasteride did not make a visible difference to Formulation B. However, when compared to Formulation A, it appeared much more well-formed and homogenous in size. This suggests that stabilisation of the particles occurred when the drug was added.

**Figure 3:**
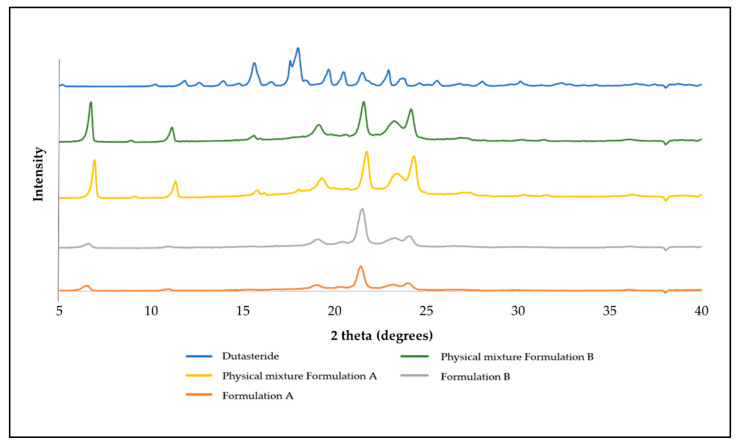
XRD results for DST (dutasteride) alone, the physical mixture of the formulation and freeze-dried Formulation A and Formulation B DST-LNP.

Based on Figure 3, the XRD pattern showed several peaks at the 2θ value of dutasteride, specifically, the peaks at 15.6 and 17.9° 2θ demonstrated the crystalline nature of dutasteride. The diffractograms of the physical mixtures were less visible at that intensity, suggesting that in the physical mixture, the drug was present in a less crystalline form. Other peaks were observed, in particular, 6.9, 11.3, 19.3, 21.7 and 24.2° 2θ; which common to both formulations, suggesting that other materials were most likely responsible for these, as they were not present in the diffractogram for the dutasteride. 

Overall, both physical mixtures appeared to be in a crystalline state. The full formulation patterns, however, showed a clear loss in the crystalline nature of the individual components, in which the peaks mentioned for the physical mixtures were significantly reduced, except for that at 21.7 2θ. This reduction was greater for Formulation A than B, suggesting that the liquid lipid played a role. The peaks showed some crystallinity but of a lower intensity, indicating that once incorporated into the formulation, the drug and the remaining component took on a more amorphous form.

#### 3.1.2. Entrapment Efficiency and Drug Loading

**Figure 4:**
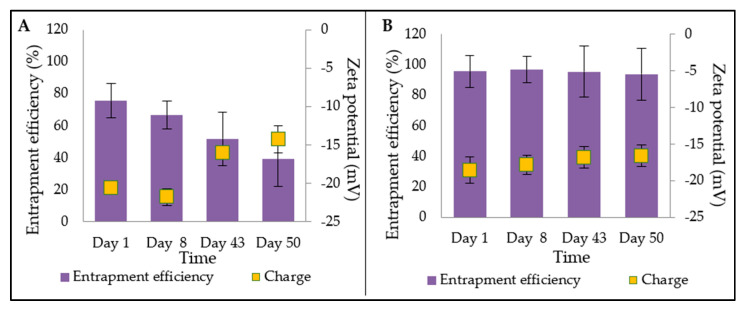
Stability test for entrapment efficiency over 50 days for Formulation A and B.

The stability of drug entrapment varied considerably between the two formulations (Figure 4). For Formulation A (Figure 4A), there was no significant difference between days 1–43. However, there was a significant difference (*p* < 0.05) between days 1 and 50. Formulation B (Figure 4B) showed no significant difference (*p* > 0.05) between days 1 and 50 for entrapment efficiency. It can be concluded that Formulation B was more physically stable based on the entrapment efficiency result throughout the study period. The entrapment efficiency was lower for Formulation A. This may have been because oleic acid, being a liquid lipid, caused imperfections in the solid lipid structure by incorporating itself into the matrix of the solid lipid [19]. These imperfections may have made it more difficult for the drug to be contained in the particle for a longer period of time, allowing greater leakage to occur. 

However, the stability of Formulation A was not significantly impacted during the first month, which means that it could still be utilised by patients over that time frame, provided that it is kept in refrigerated conditions. This issue can be potentially resolved by freeze-drying the optimised formulation and reconstituting it in water before use. Alternatively, further optimisation of the formulation through the addition of more surfactant may also improve stability. The added advantage of Formulation A is the additional anti-androgenic properties associated with oleic acid [17,20,21], which is itself an active ingredient itself. This means that patients may observe a faster response from Formulation A.

**Figure 5:**
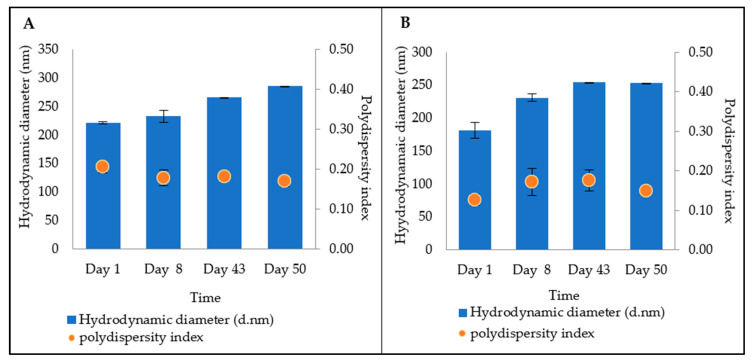
Stability test for particle size distribution over 50 days for Formulation A (**A**) and B (**B**).

From Figure 5, for both formulations, the results showed that over time, the hydrodynamic size increased slightly. However, on day 50, the size was still below 300 nm, which means that the preparation could be possible to penetrate the skin, as the spironoltactone loaded nanostructured lipid carriers in the size range of 216–834 nm have previously been applied for follicular delivery [22]. The pattern of increase in size showed a variation, namely, Formulation A showed nonsignificant initial increase (*p* < 0.05) until day 8, that proceeded to become significant by days 43 and 50. For Formulation B, an initial significant increase (*p <* 0.05) in size was observed until day 8, which thereafter increased more slowly. The polydispersity index decreased for Formulation A, which means that that level of variance of particle size diminished, leading to a more homogenous formulation. In contrast, for Formulation B, no significant changes in the polydispersity index were observed between day 43 and day 50, suggesting that the particles had reached a stable condition.

### 3.2. In Vitro Drug Release

**Figure 6:**
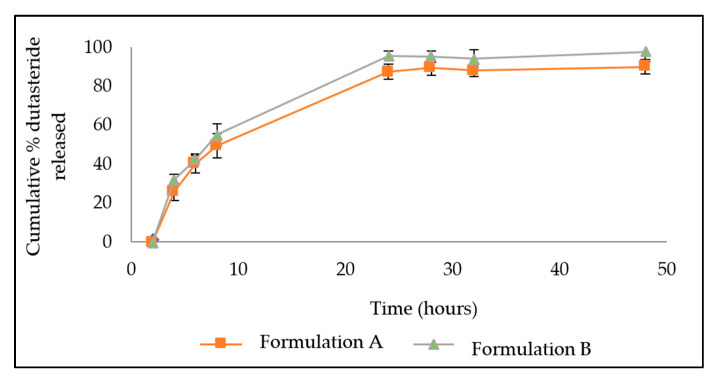
Dutasteride release from DST-LNP for Formulation A and B over 48 h.

The release studies involved observations of how much drug passed through a cellulose membrane over a period of 48 h. Both formulations showed slow release from the LNP over 48 h (Figure 6). Formulation B released a higher amount of drug at all time points, reaching 97.5% by hour 48, whereas formulation A released 90%. 

### 3.3. In Vitro Permeation Study 

**Figure 7:**
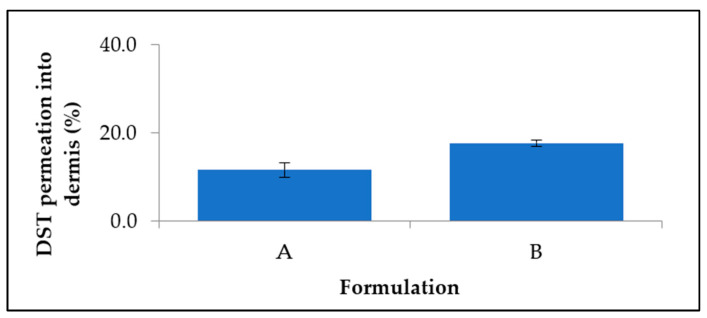
Comparison of dutasteride permeation from DST-LNP of Formulation A and B over 48 h.

In vitro permeation studies using porcine ear skin were conducted to mimic human skin. Porcine ear is a good in vitro model for human skin, as it presents a similar penetration for topically applied substances. The receptor chamber of the Franz diffusion cell did not contain any dutasteride when tested for permeation by HPLC. This suggests that the drug may not penetrate deep enough to reach the capillaries, which has the advantage of reducing systemic absorption and side-effects. Previous attempts to formulate finasteride into LNP showed similar results, i.e., negligible quantities of finasteride permeated into the receptor, although the content in the dermis itself was not analysed [20]. For both samples, <20% of the drug permeated into the dermis after 48 h (Figure 7). The rest of the drug was localised in the donor compartment and the stratum corneum site. Formulation A had an average permeation of 11.5 ± 1.7% with significant difference with Formulation B, which had 17.7 ± 0.7% permeation, suggesting that dutasteride delivered in a smaller quantity of liquid lipid achieved higher permeation into the dermis. As suggested in a previous study, the improvement of skin permeation effectiveness is influenced by both drug partitioning between the skin and vehicles, and drug–vehicle interactions. Solid lipids which form highly crystalline particles with a perfect lattice might result in drug expulsion [21]. 

It has previously been reported that dye encapsulated in 320 nm nanoparticles travels much deeper into hair follicles if a massage is applied, compared to the dye in nonparticle form [23]. The study found that, the hair follicles behave as a long-term reservoir for topically applied substances, allowing the storage of such substances for up to 10 days, compared to only 4 days for nonparticle forms [23]. Though it was not possible to apply the massaging technique in this study, massaging may lead to deeper penetration. This could be tested with further studies. Hair follicles are essentially found in the dermis of the skin; having this kind of particle size in our case could be used for follicular targeting. It has been found that particles sized between 230–300 nm are able to target this site, meaning that the formulation should be able to penetrate the upper part of the skin, as was notably demonstrated by Formulation A. Formulation B contained of particles of a smaller size than Formulation A, which means that it would also be likely to penetrate deeper in the skin.

## 4. Conclusions

Male pattern baldness is a condition which is becoming increasingly prevalent. The treatment available in the market are very limited, and none of which utilises dutasteride as an anti-androgenic molecule for promoting hair growth. Dutasteride, a potent 5α reductase enzyme inhibitor, has shown strong potential in preventing hair loss, but limited research has been done on this subject. In this study, dutasteride-loaded LNPs were successfully produced using the emulsification–ultrasonication method. Constituents that possess anti-androgenic activity themselves were utilised to add potency to the preparation. A comparison was successfully made between oleic and stearic acid as the liquid and solid lipid, respectively. Formulations A and B resulted in a size range of 180–230 nm, with a charge that was high enough to ensure good electrostatic stabilisation. High entrapment efficiency was achieved for both compositions. Even after 50 days of stability testing, the particle size remained below 300 nm, indicating good physical stability in storage. Morphological investigations showed that all nanoparticles exhibited a spherical shape. Release studies demonstrated a >90% release for both compositions after 48 h, and showed a prolonged release of the drug from the solid lipid matrix of the nanoparticles. This slow release profile is beneficial for drugs with a prolonged time of action, as it avoids the need for reapplication. No drug was found to permeate the skin, which reflects good suitability for topical application, avoiding systemic side effects. These results suggest that the proposed formulation presents several characteristics which are novel, including its stability and penetration. As such, it presents key findings on the dermal delivery of anti-alopecia active compounds.
